# Editorial Comment: Solifenacin treatment after intradetrusor injections with botulinum toxin in patients with neurogenic detrusor overactivity

**DOI:** 10.1590/S1677-5538.IBJU.2022.06.02

**Published:** 2022-11-01

**Authors:** Marcio Augusto Averbeck

**Affiliations:** 1 Unidade de Videourodinâmica Hospital Moinhos de Vento Chefe de Neuro-Urologia Porto Alegre RS Brasil Chefe de Neuro-Urologia, Unidade de Videourodinâmica, Hospital Moinhos de Vento. Porto Alegre, RS, Brasil

Irina Ciofu ^1^, Iuliana Ceausu ^1^, Narcis Marian Chirca ^1^, Cristian Persu ^1^

**Am J Ther. 2022 Jun 21. Online ahead of print**

**DOI: 10.1097/MJT.0000000000001531 | ACCESS: 35731251**

## COMMENT

This prospective study evaluated whether adding solifenacin to the intradetrusor injection of botulinum toxin A (BoNT) could boost the effect of BoNT in patients with neurogenic detrusor overactivity (NDO) due to multiple sclerosis or spinal cord injury refractory to antimuscarinics alone (1). Thirty-nine patients who achieved total continence after BoNT injections were included in the analysis (group A: BoNT injections; group B: BoNT + solifenacin) and were followed for a minimum of 24 months. Data from urodynamic testing and questionnaire assessments before and 3 months after injections and reinjections were gathered.

Reinjection was needed after a mean 8.2 months for group A and 11.7 months for group B. Patients receiving solifenacin also presented greater OABq score improvement (A = 17.25 ± 5.07, B = 20.44 ± 4.51, P = 0.0485), as well as maximum bladder capacity (A = 11.05 ± 7.04 mL, B = 19.39 ± 6.43 mL, P = 0.0005). However, differences in Pdet change (A = 51.72 ± 16.57 cmH2O, B = 50.80 ± 16.33 cmH2O, P = 0.7635) and post-void residual change (A = 17.67 ± 12.63 mL, B = 12.30 ± 8.32 mL, P = 0.126) were not statistically significant.

Authors concluded that adding solifenacin to BoNT improved patient satisfaction and increased the interval between reinjections. Nevertheless, this is a non-randomized trial, which lacked a placebo control group. Further well-designed studies (e.g. RTCs) are still warranted before definitive conclusions may be drawn concerning the role of adding antimuscarinics to patients receiving BoNT to treat NDO.
